# Analysis of influencing factors for cognitive impairment in children with self-limited epilepsy with centrotemporal spikes

**DOI:** 10.3389/fneur.2026.1681841

**Published:** 2026-01-15

**Authors:** Huifang Yan, Chen Chen, Sisi Geng

**Affiliations:** Harrison International Peace Hospital, Hengshui, China

**Keywords:** clinical intervention, cognitive function, pediatric patients, risk factors, self-limited epilepsy with centrotemporal spikes

## Abstract

**Objective:**

This study aimed to analyze the influencing factors of cognitive impairment in children with Self-limited Epilepsy with Centrotemporal Spikes (SeLECTS) to provide a scientific basis for clinical intervention.

**Methods:**

We retrospectively analyzed the clinical data of children diagnosed with SeLECTS at our hospital between January 2020 and December 2024. Intelligence scale assessments were collected, and patients were divided into cognitive dysfunction and non-cognitive dysfunction groups based on the results. Data on age, seizure frequency, spike peak voltage, electroencephalogram (EEG) discharge index, and laboratory indicators were obtained for both groups. The predictive value of EEG and laboratory indicators for cognitive impairment in SeLECTS patients was evaluated using Receiver Operating Characteristic (ROC) curve analysis and multivariate logistic regression.

**Results:**

A total of 106 SeLECTS children were included, of whom 35 (33.02%) exhibited cognitive dysfunction. Compared to the non-cognitive dysfunction group, the cognitive dysfunction group had a younger age of onset and higher levels of interleukin-6 (IL-6), tumor necrosis factor-*α* (TNF-α), neuron-specific enolase (NSE), spike peak voltage, and a greater proportion with a discharge index >10% (*p* < 0.05). The areas under the ROC curve (AUC) for IL-6, TNF-α, and NSE in assessing cognitive impairment were 0.842, 0.833, and 0.855, respectively. The combined AUC of these three markers was 0.928, which was significantly higher than that of IL-6 (*Z* = 2.297, *p* = 0.022), TNF-α (*Z* = 2.296, *p* = 0.022), and NSE (*Z* = 2.108, *p* = 0.035) individually. The AUC values for spike peak voltage and discharge index were 0.620 and 0.637, respectively, while their combined AUC was 0.696. Elevated IL-6, TNF-α, and NSE levels were identified as risk factors for cognitive impairment in SeLECTS children (*p* < 0.05), whereas older age of onset was a protective factor (*p* < 0.05).

**Conclusion:**

IL-6, TNF-α, NSE, spike peak voltage, discharge index, and age of onset are associated with cognitive impairment in SeLECTS children and may serve as valuable potential biomarkers, providing insights for early intervention.

## 1 Introduction

Self-limited Epilepsy with Centrotemporal Spikes (SeLECTS) is the most common focal epilepsy syndrome in childhood, typically presenting between the ages of 4 and 15 years (1). Seizures in SeLECTS exhibit distinct characteristics, with most episodes occurring during sleep, particularly at sleep onset or before awakening. The manifestations primarily include focal motor and sensory seizures involving the face and oropharyngeal region, such as unilateral mouth deviation, facial twitching, perioral numbness, and hypersalivation. In some cases, secondary generalized tonic–clonic seizures may occur (2).

Although SeLECTS is generally considered a benign epilepsy syndrome with a favorable seizure prognosis and no underlying structural abnormalities, growing evidence indicates that affected children may experience specific cognitive and behavioral impairments during the active phase of epilepsy. These deficits predominantly involve attention, memory, and executive function (3). Such cognitive dysfunction significantly impacts learning, daily living, and social interactions, potentially hindering long-term developmental outcomes. Therefore, in addition to seizure control, close monitoring of cognitive function is essential in the management of SeLECTS.

The exact mechanisms underlying cognitive impairment in SeLECTS remain incompletely understood and are likely multifactorial. While recent studies have made progress in characterizing these deficits, most investigations have relied on limited neuropsychological measures (4). To address this gap, the present study employs a multidimensional assessment of cognitive function, incorporating demographic, serological, and electroencephalographic (EEG) parameters to comprehensively analyze risk factors for cognitive impairment in SeLECTS. The findings may facilitate early identification of high-risk patients, enabling timely interventions to improve cognitive outcomes and quality of life.

## 2 Materials and method

### 2.1 Study subjects

This study retrospectively analyzed the clinical data of children with SeLECTS who were treated at our hospital from January 2020 to December 2024. The inclusion criteria were as follows: (1) age range: 6–16 years; (2) diagnosis of SeLECTS confirmed according to the *Clinical Guidelines for Epilepsy (2023 Edition)*; (3) normal interictal EEG background with spikes or spike-and-wave complexes observed in the central and temporal regions, either unilaterally, bilaterally, or alternately, and increased epileptiform discharges during sleep; (4) disease duration of at least 6 months with a minimum of two seizures; (5) exclusion of other neurological disorders (e.g., brain injury, congenital malformations) via neuroimaging; (6) no current use of medications that may significantly impair cognitive function (e.g., benzodiazepines, topiramate); (7) the time interval between the last epileptic seizure and the detection of IL-6, TNF-α, and NSE was at least 2 weeks. The exclusion criteria were: (1) presence of other epilepsy types or syndromes (e.g., Lennox–Gastaut syndrome); (2) structural brain lesions (e.g., tumors, post-encephalitic sequelae); (3) IQ < 70 or prior diagnosis of intellectual disability or global developmental delay; (4) severe systemic diseases (e.g., metabolic disorders, genetic syndromes); (5) psychiatric disorders (e.g., autism) or sensory impairments (hearing/visual abnormalities); (6) use of antidepressants, sedatives, or other neuroactive drugs within the past 3 months; (7) inability to cooperate with neuropsychological assessments or parental refusal to participate. This study was approved by the hospital’s ethics committee. After rigorous screening based on the inclusion and exclusion criteria, a total of 106 children with SeLECTS were enrolled in this study.

### 2.2 Cognitive function assessment and grouping

The uniform time point for testing was defined as “within 1 week after the child was enrolled, during a stable period of seizure control (characterized by no seizure episodes within the recent 2 weeks, and no occurrence of status epilepticus or electrical status epilepticus during slow-wave sleep (ESES)). The Chinese revised version of the Wechsler Intelligence Scale for Children (WISC-IV) (5) was used to evaluate the participants’ verbal comprehension index (VCI), perceptual reasoning index (PRI), working memory index (WMI), processing speed index (PSI), and full-scale intelligence quotient (FIQ). After enrollment screening, each child underwent one-on-one testing administered by a child neuropsychological assessor who had received standardized training on the WISC-IV. The assessments were conducted in a quiet and non-distracting environment within the neuropsychological evaluation room of our hospital. Each test session lasted 60–90 min, ensuring that the evaluations were completed under stable physical and mental conditions for every participant.

A normal FIQ level was defined as 90–109. Children with an FIQ < 70 were classified as having intellectual disability (after excluding measurement errors), while scores of 80–89 and 70–79 were considered below average and borderline, respectively. Participants were assigned to the cognitive dysfunction group if they had an FIQ < 80 or an FIQ ≥ 80 but with at least one subscale score (VCI, PRI, WMI, or PSI) < 80. Conversely, those with an FIQ ≥ 80 and all subscale scores (VCI, PRI, WMI, and PSI) ≥ 80 were classified into the non-cognitive dysfunction group.

### 2.3 Data collection

Clinical data of the pediatric patients were collected by reviewing electronic medical records. (1) Demographic and baseline characteristics, such as age, sex, parental education level, age at disease onset, seizure frequency, seizure type, and usage of antiepileptic drugs. (2) Laboratory parameters, including interleukin-6 (IL-6), tumor necrosis factor-*α* (TNF-α), and neuron-specific enolase (NSE). In this study, the time points for detecting IL-6, TNF-α, and NSE were aligned with those for cognitive function assessment. (3) EEG indicators, comprising the discharge localization during seizures, spike peak voltage (potential difference from baseline to peak), and discharge index. The discharge index was calculated as the duration of epileptiform discharges during sleep divided by the total duration of non-rapid eye movement (NREM) sleep. All procedures were performed by neurophysiologists with over 5 years of clinical experience.

### 2.4 Statistical analysis

Statistical analyses were performed using SPSS 26.0 software. Continuous variables conforming to a normal distribution were presented as mean ± standard deviation (SD), and comparisons between groups were conducted using independent samples *t*-tests. Categorical variables were described as counts (percentages), and intergroup differences were assessed using the chi-square (*χ*^2^) test. Binary logistic regression analysis was employed to identify influencing factors associated with cognitive impairment in children with SeLECTS. The predictive performance of laboratory and EEG indicators for cognitive impairment in SeLECTS patients was evaluated using receiver operating characteristic (ROC) curve analysis. The area under the curve (AUC) was interpreted as follows: AUC < 0.7 indicated low predictive accuracy, 0.7–0.79 suggested moderate predictive ability, 0.8–0.9 denoted high predictive performance, and > 0.9 reflected excellent predictive power. A two-tailed *p*-value < 0.05 was considered statistically significant.

## 3 Results

### 3.1 Comparison of clinical characteristics between children with and without cognitive dysfunction

A total of 106 children with SeLECTS were enrolled in this study, among whom 35 (33.02%) exhibited cognitive dysfunction. Compared to the non-cognitive dysfunction group, children with cognitive dysfunction had a significantly younger age at onset, along with higher levels of IL-6, TNF-α, and neuron-specific enolase (NSE), as well as greater spike peak voltage and a higher proportion of patients with a discharge index >10% (*p* < 0.05, Table 1).

**Table 1:** Comparison of clinical characteristics between children with and without cognitive dysfunction.

Parameter	Cognitive dysfunction group (*n* = 35)	Non-cognitive dysfunction group (*n* = 71)	*t*/ $\chi^{2}$	*p*
Sex [*n* (%)]			0.797	0.372
Male	19 (54.3)	32 (45.1)		
Female	16 (45.7)	39 (54.9)		
Age (years)	10.26 ± 1.69	10.68 ± 2.00	1.066	0.289
Parents’ education level [*n* (%)]			2.013	0.156
High school and below	13 (37.1)	17 (23.9)		
College and above	22 (62.9)	54 (76.1)		
Age of onset (years)	6.26 ± 1.25	7.31 ± 1.46	3.659	<0.001
Type of seizure [*n* (%)]			0.597	0.440
Focal seizure	21 (60.0)	48 (67.6)		
Generalized seizure	14 (40.0)	23 (32.4)		
Frequency of seizures [*n* (%)]			1.506	0.220
>5 times/year	14 (40.0)	20 (28.2)		
≤5 times/year	21 (60.0)	51 (71.8)		
IL-6 (μg/L)	34.35 ± 7.13	25.11 ± 5.66	7.241	<0.001
TNF-α (μg/L)	40.57 ± 6.40	31.78 ± 6.09	6.874	<0.001
NSE (μg/L)	14.92 ± 2.47	11.52 ± 1.95	7.727	<0.001
Site of discharge [*n* (%)]			0.581	0.446
Central temporal region	31 (88.6)	66 (93.0)		
Outside the central temporal region	4 (11.4)	5 (7.0)		
Spike peak voltage (μV)	14.77 ± 2.44	13.51 ± 2.90	2.219	0.029
Discharge index [*n* (%)]			8.859	0.003
>10%	16 (45.7)	13 (18.3)		
≤10	19 (54.3)	58 (81.7)		

### 3.2 Evaluation value of laboratory indicators for cognitive impairment in children with SeLECTS

**Figure 1:**
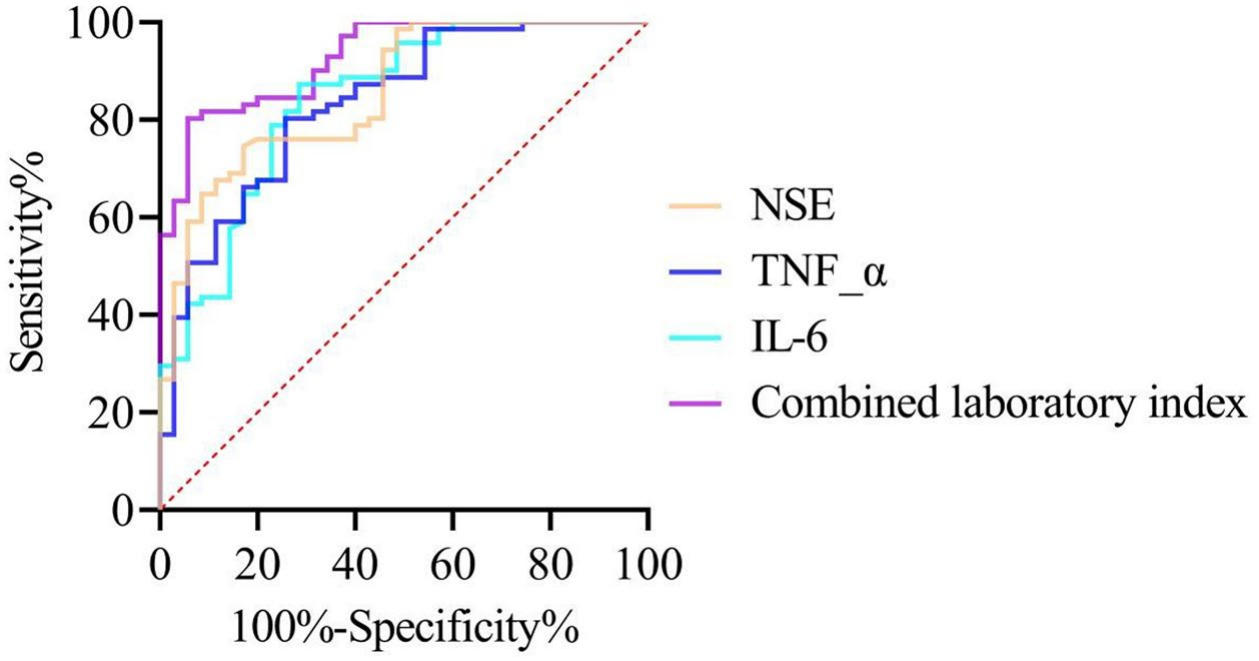
ROC curve analysis of laboratory indicators for assessing cognitive impairment in children with SeLECTS.

ROC analysis revealed that the AUC values of IL-6, TNF-α, and NSE for assessing cognitive impairment in children with SeLECTS were 0.842, 0.833, and 0.855, respectively. These three indicators were incorporated into a logistic regression model, and a combined laboratory index was derived based on the regression coefficients: Combined laboratory index = −30.295 + 0.289 × IL-6 + 0.314 × TNF-α + 0.746 × NSE. The results demonstrated that the combined index achieved an AUC of 0.928, which was significantly higher than that of IL-6 (*Z* = 2.297, *p* = 0.022), TNF-α (*Z* = 2.296, *p* = 0.022), and NSE (*Z* = 2.108, *p* = 0.035). The sensitivity and specificity of the combined index were 94.29 and 80.28%, respectively (Table 2; Figure 1).

**Table 2:** Evaluation value of laboratory indicators for cognitive impairment in children with SeLECTS.

Indicators	AUC	Cut-off	95%*CI*	Sensitivity (%)	Specificity (%)	Youden’s index	*p*
IL-6	0.842	31.73	0.760 ~ 0.923	71.43 (25/35)	87.32 (62/71)	0.588	<0.001
TNF-α	0.833	37.0	0.752 ~ 0.912	74.29 (26/35)	80.28 (57/71)	0.546	<0.001
NSE	0.855	12.64	0.781 ~ 0.929	82.86 (29/35)	74.65 (53/71)	0.575	<0.001
Combined laboratory index	0.928	−1.22	0.880 ~ 0.975	94.29 (33/35)	80.28 (57/71)	0.746	<0.001

### 3.3 Evaluation value of EEG indicators for cognitive impairment in children with SeLECTS

**Figure 2:**
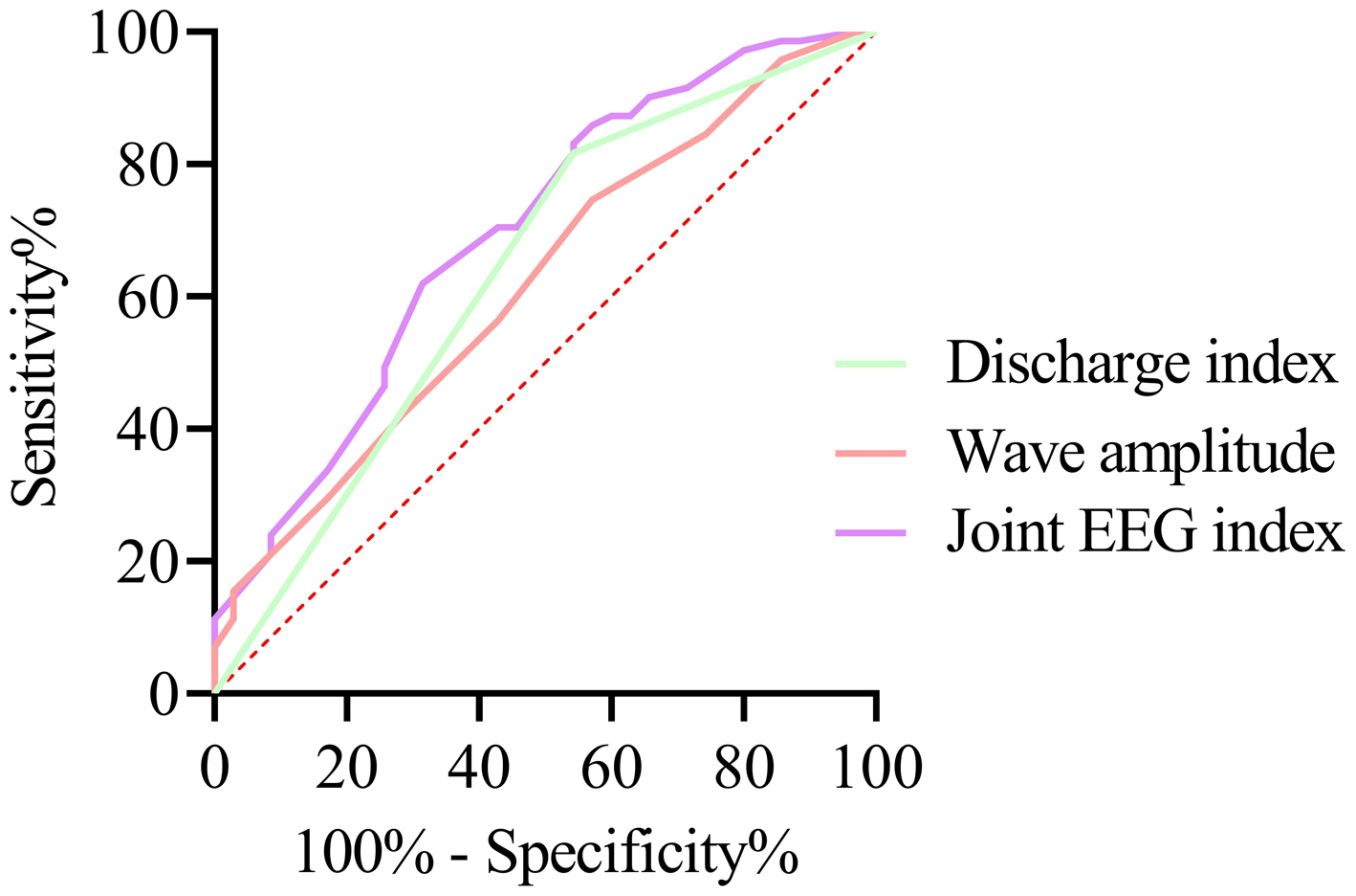
ROC curve analysis of EEG indicators for assessing cognitive impairment in children with SeLECTS.

The ROC analysis revealed that the AUC values for spike peak voltage and discharge index in assessing cognitive impairment in children with SeLECTS were 0.620 and 0.637, respectively. These two EEG parameters were incorporated into a logistic regression model, and a combined EEG indicator was derived based on the regression coefficients: Combined EEG indicator = −3.652 + 0.179 × spike peak voltage + 1.343 × discharge index. The results demonstrated that the combined EEG indicator achieved an AUC of 0.696, with a sensitivity of 68.57% and a specificity of 61.97%, as detailed in Table 3 and Figure 2.

**Table 3:** Evaluation value of EEG indicators for cognitive impairment in children with SeLECTS.

Indicators	AUC	Cut-off	95%*CI*	Sensitivity (%)	Specificity (%)	Youden’s index	*p*
Spike peak voltage	0.620	15	0.507 ~ 0.732	42.86 (25/35)	74.65 (62/71)	0.175	0.036
Discharge index	0.637	0	0.520 ~ 0.754	45.71 (26/35)	81.69 (57/71)	0.274	0.005
Combined EEG indicator	0.696	−0.97	0.588 ~ 0.804	68.57 (29/35)	61.97 (53/71)	0.305	<0.001

### 3.4 Multivariate analysis of cognitive impairment in children with SeLECTS

Using the presence of cognitive impairment as the dependent variable (cognitive impairment = 1, no impairment = 0), and incorporating the following independent variables—age at onset, IL-6, TNF-α, NSE levels, spike peak voltage (all as continuous variables), and discharge index (> 10% = 1, ≤ 10% = 0)—into a logistic regression model, the analysis revealed that IL-6, TNF-α, and NSE were significant risk factors for cognitive impairment in children with SeLECTS (*p* < 0.05, Table 4). In contrast, age at onset served as a protective factor against cognitive impairment (*p* < 0.05, Table 4).

**Table 4:** Multivariate analysis of cognitive impairment in children with SeLECTS.

Factor	B	SE	*Wald*	*OR*	95%CI	*p*
IL-6	0.284	0.088	10.492	1.328	1.118 ~ 1.576	0.001
TNF-α	0.332	0.100	11.075	1.394	1.146 ~ 1.695	0.001
NSE	0.800	0.252	10.118	2.227	1.360 ~ 3.646	0.001
Age of onset	−0.797	0.395	4.068	0.451	0.28 ~ 0.978	0.044

## 4 Discussion

Benign Childhood Epilepsy with Centrotemporal Spikes (BECTS) was previously considered a “benign” epilepsy syndrome. However, studies have revealed that BECTS is associated with cognitive dysfunction (6), particularly in memory, attention, and executive function. Some research even suggests that cognitive impairments in BECTS may be generalized. Consequently, the 2022 ILAE classification of epilepsy syndromes no longer uses the term “benign,” replacing it with “Self-Limited Epilepsy with Centrotemporal Spikes” (SeLECTS). Previous studies have primarily focused on domain-specific cognitive deficits in children with SeLECTS (7, 8), yet systematic investigations examining the relationship between cognitive function and clinical/EEG characteristics remain scarce. This study aims to explore the clinical factors, laboratory indicators, and EEG parameters in children with SeLECTS to identify potential influencing factors associated with cognitive impairment, thereby providing insights for early intervention and disease management.

The present study found that the cognitive dysfunction group exhibited a younger age at seizure onset, and multivariate logistic regression analysis identified age at onset as a protective factor against cognitive impairment in children with SeLECTS. The developing brain possesses remarkable plasticity during childhood. An earlier age at seizure onset implies that epileptic discharges occur during a critical period of rapid brain development, where frequent abnormal electrical activity may disrupt normal neuronal migration, differentiation, and synaptogenesis, ultimately leading to aberrant structural and functional brain development (9). Studies have demonstrated that early-life seizures can cause neuronal damage and gliosis in cognition-related regions such as the hippocampus, resulting in impairments in memory, executive function, and other cognitive domains (10, 11). In contrast, children with later seizure onset may have achieved relatively more mature brain development prior to epilepsy onset, consequently exhibiting greater resilience to epileptic activity and milder cognitive sequelae. Therefore, clinicians should pay particular attention to cognitive development in SeLECTS patients with younger age at onset and implement early interventions when necessary.

This study revealed that serum levels of IL-6, TNF-α, and NSE were significantly elevated in children with cognitive dysfunction compared to those without, and all three biomarkers were identified as risk factors for cognitive impairment in children with SeLECTS. The pathogenesis of SeLECTS has not been fully elucidated, although accumulating evidence suggests that chronic inflammatory responses play a pivotal role in disease progression and cognitive impairment (12). Epileptiform discharges can activate microglia and astrocytes in the brain, leading to the release of pro-inflammatory cytokines such as IL-6 and TNF-α. These cytokines contribute, directly or indirectly, to damage in cognition-related brain regions—including the hippocampus and prefrontal cortex—through mechanisms such as disruption of blood–brain barrier integrity, induction of neuronal excitotoxicity, and interference with synaptic plasticity (13). Furthermore, inflammatory cytokines activate microglia, exacerbating neuroinflammation, neuronal damage, synaptic loss, and vascular pathology, ultimately contributing to cognitive decline (14). NSE is a cytoplasmic enzyme specifically expressed in neurons and neuroendocrine cells. Under normal conditions, its serum concentration is extremely low. However, when neuronal damage or necrosis occurs, NSE is released into the bloodstream, making it a sensitive biomarker for the extent of neuronal injury (15). Moreover, serum NSE levels are negatively correlated with the severity of cognitive impairment—higher NSE levels indicate more severe neuronal damage and a greater risk of cognitive dysfunction (16). In this study, the AUC for IL-6, TNF-α, and NSE in predicting cognitive impairment in SeLECTS patients all exceeded 0.7, indicating their significant predictive value. The combined assessment of these three biomarkers yielded an AUC of 0.928, surpassing individual measurements, with high sensitivity and specificity. This suggests that a multi-parameter approach enhances diagnostic accuracy in evaluating cognitive impairment risk in SeLECTS patients. Each biomarker reflects distinct pathophysiological processes: IL-6 and TNF-α indicate the severity of neuroinflammation, while NSE quantifies neuronal injury. Their combined analysis provides complementary insights, improving diagnostic reliability. In clinical practice, a multi-marker panel may facilitate early diagnosis and intervention for cognitive impairment in SeLECTS patients.

On neuroimaging examinations, children with SeLECTS typically lack distinctive positive findings. However, EEG reveals characteristic centrotemporal spikes, which are often more pronounced during sleep. In some patients, near-continuous discharges may occur during non-rapid eye movement (NREM) sleep, a condition termed electrical status epilepticus during sleep (ESES) (17, 18). The spike peak voltage and discharge index reflect abnormal neuronal discharges in the brain. Higher spike peak voltages and discharge indices may indicate increased seizure frequency and severity, thereby elevating the risk of cognitive impairment (19, 20). In this study, the cognitive dysfunction group exhibited a higher proportion of patients with spike peak voltages and discharge indices >10%. However, the predictive value of spike peak voltage and discharge index—either individually or in combination—for assessing cognitive impairment in SeLECTS patients was limited. This observation may be attributed to the influence of multiple factors on EEG parameters, resulting in significant interindividual variability. Additionally, the complexity of epileptic seizures imposes limitations on relying solely on spike peak voltage and discharge index to evaluate cognitive dysfunction. Nevertheless, these metrics may still provide supplementary clinical insights, and their integration with other indicators could facilitate a more comprehensive assessment of cognitive function in affected children.

However, this study still has several limitations. As a single-center retrospective analysis, this study is limited by a relatively small sample size comprising exclusively Han Chinese children, with no inclusion of patients from diverse ethnic backgrounds to assess the potential influence of racial factors. This constraint restricts the generalizability of our findings. Future international collaborative studies involving multi-ethnic cohorts and multiple centers are warranted to validate and extend the conclusions drawn from the present research. Furthermore, the study only examined a limited number of inflammatory factors, neuronal injury markers, and EEG parameters, potentially overlooking other factors associated with cognitive impairment. Future research should expand the sample size and incorporate additional potential influencing factors to enhance the understanding of determinants contributing to cognitive dysfunction in children with SeLECTS. Gender, as an important biological variable, may play a role in the long-term cognitive prognosis of epilepsy. In this study, the chi-square test was used to compare the gender distribution between the two groups of children. The results showed no statistically significant difference, which did not further reveal the potential effect of gender on the long-term dynamic changes in cognitive function. Future studies are needed to further explore the complex relationships among gender, sex hormone levels, and the cognitive developmental trajectory in children with SeLECTS.

In conclusion, younger age of onset, elevated levels of IL-6, TNF-α, and NSE were identified as significant risk factors for cognitive impairment in children with SeLECTS. Clinicians should monitor these biomarkers to facilitate early identification of high-risk patients. Timely interventions—including antiepileptic therapy, anti-inflammatory treatment, and cognitive rehabilitation—may improve cognitive outcomes in this population.

## Data Availability

The original contributions presented in the study are included in the article/Supplementary material, further inquiries can be directed to the corresponding author.
